# The Impact of Recovery of Visuo-Spatial Neglect on Motor Recovery of the Upper Paretic Limb after Stroke

**DOI:** 10.1371/journal.pone.0100584

**Published:** 2014-06-20

**Authors:** Tanja C. W. Nijboer, Boudewijn J. Kollen, Gert Kwakkel

**Affiliations:** 1 Utrecht University, Experimental Psychology, Utrecht, the Netherlands; 2 Rudolf Magnus Institute of Neuroscience and Center of Excellence for Rehabilitation Medicine, University Medical Center Utrecht and de Hoogstraat Rehabilitation Center, Utrecht, the Netherlands; 3 Department of General Practice, University of Groningen, University Medical Center Groningen, Groningen, the Netherlands; 4 VU University Medical Center, Department of Rehabilitation Medicine, Research Institute MOVE, Amsterdam, the Netherlands; 5 Department of Neurorehabilitation, Centre of Rehabilitation and and Rheumatology READE, Amsterdam, the Netherlands; University of Rome, Italy

## Abstract

The aim of the current study was to investigate the longitudinal relationship between improvements of synergism and strength of the upper paretic limb and severity of visuo-spatial neglect during the first 52 weeks post-stroke. The longitudinal association between severity of VSN and motor impairment using Fugl Meyer motor score and Motricity Index of the arm was measured in an intensive repeated measurement design including 18 measurement sessions for each subject. Neglect was assessed using the letter cancellation test applied in a prospective cohort of 101 ischemic, first-ever, hemispheric stroke patients. All time-dependent measures were taken weekly, starting within 14 days post-stroke. From week 10 to 20 biweekly measurements are obtained. The longitudinal relationship of (bi)weekly time on improvement of motor functions and severity of neglect was investigated using random coefficient analysis and trend analyses. Fifty-one of the 101 stroke patients showed neglect at stroke onset. Less improvement of synergism and strength of the upper paretic limb was associated with more severe neglect. This association was most pronounced in the first 10 weeks post-stroke. The seemingly suppressive effect of neglect on upper-limb motor recovery appears to take place mainly during spontaneous neurological recovery of first 10 weeks post-stroke. This finding suggests that damage to large-scale white matter tracts of especially the perceptual-attention networks suppress recovery of other networks at distance in the brain suggesting a common underlying mechanism.

## Introduction

Visuospatial neglect (VSN) is a frequent post-stroke disorder [1], [2], where patients demonstrate impaired awareness for contralesional stimuli. VSN is linked to poor motor recovery, higher disability, poor responses to rehabilitation services [3], yet the time course of suppressive effects of VSN are largely unknown [4] due to lack of prospective cohort studies satisfying the key methodological criteria for prognostic research according to the STROBE statement [5].

The aim of the current study is to investigate the time course of suppressive effects of VSN on the severity and time course of synergistic motor control (FM-arm) and motor strength (Motricity Index-arm (MI-arm)) of the upper paretic limb in the first year post-stroke are investigated. Progress of time alone, as a reflection of spontaneous neurological recovery, is only statistically significant for strength, synergism and VSN within the first 10 weeks post-stroke [6], [7]. Clinically, an early observed recovery pattern of synergic-dependent motor control (Fugl-Meyer (FM) motor scores [8], [9], [10] is often interpreted as reflecting ‘true neurological repair’ by which patients regain their ability to control the different degrees of freedom in the paretic upper limb [10], [11], [12]. VSN severity was taken as a time-dependent predictor to investigate the impact of VSN recovery on motor recovery. We hypothesize that VSN not only is associated with more motor impairment at stroke onset, but also with reduced motor improvement in the first months post-stroke. Additionally, these suppressive effects will be mainly restricted to the same time-window where spontaneous neurological recovery takes place. These effects of VSN on the time course of motor recovery will be comparable for FM-arm and MI-arm, due to a common underlying suppressive mechanism affecting motor networks that gradual alleviate in time [13].

## Materials and Methods

### Participants

101 stroke patients (mean age: 65 years (SD = 12) participated. Data from these patients were published before [7], [14], [15]. Inclusion criteria were: (1) aged between 30 and 80 years; (2) ischemic, first-ever, stroke, involving medial or anterior cerebral arteries as revealed by CAT or MRI; (3) inability to walk at first assessment; (4) no complicating medical history such as cardiac, pulmonary, or orthopedic disorders; (5) no severe deficits in communication, understanding, and memory; (6) written or verbal informed consent and sufficient motivation to participate. The Mini-Mental State Examination (MMSE; [16]) was used to screen cognitive impairment. Only patients with a score of >24 were included in the trial. A speech therapist assessed the ability to communicate and accepted a cut-off point of the 50th percentile corrected for age on the Dutch Foundation Aphasia Test [17].

Of the 101 stroke patients (Table 1), 51 showed VSN in week 1, as measured with a letter cancellation test. None of the patients received training to ameliorate VSN.

**Table 1:** Demographical and stroke characteristics per group (VSN versus non-VSN).

Clinical variables	Results VSN (SD)	Results Non-VSN (SD)
Group size	51	50
Age in years	66.59 (10.165)	65.10 (10.994)
Sex (male-female)	51%–49%	62%–38%
Time post-stroke in days	7.96 (3.098)	8.30 (2.597)
Hemisphere of stroke (Left/Right; n)	9/42	34/16
Site of stroke		
* TACI*	88.2%	32%
* PACI*	11.8%	54%
* LACI*	0%	14%
Treatment		
* Airsplint*	37.3%	36%
* Arm*	35.3%	30%
* Leg*	27.5%	27.5%
MMSE (0–30)	25.73 (2.270)	26.91 (2.589)
Barthel Index at start (0–20)	3.41 (2.153)	5.63 (2.785)
Sensory deficit (TFT)		
* No deficit (N = )*	8	21
* Within thumb area (n = )*	18	19
* Following the arm (n = )*	14	8
* Unable (n = )*	10	2
Fugl Meyer Arm	6.81 (7.06)	10.70 (9.66)
Fugl Meyer Leg	8.86 (7.18)	12.11 (6.71)
Motricity Index Arm (0–100)	6.26 (15.79)	14.81 (22.97)
Motricity Index Leg (0–100)	13.58 (18.90)	25.29 (21.95)

### Procedure

The research protocol was implemented within 14 days after stroke onset. Final outcome was defined at 52 weeks after stroke. Each entire testing procedure took 45–75 minutes, depending on the level of disability [14].

### Outcome measures

As most improvements were expected to emerge in de first months post-stroke, weekly measurements were done during the initial ten weeks, followed by biweekly measurements until the 20^th^ week. Thereafter, follow-up measurements were performed at 26, 38 and 52 weeks. All outcome measures were obtained during these sessions.

The patient's medical record was reviewed. The following admission data were captured: age, sex, time post-stroke, site of stroke, MMSE, Barthel Index, Letter Cancellation Test, sensory deficit in the arm (Thumb-Finding Test (TFT); 0 to 3 points) as a part of the Orpington Prognostic Score [18], [19]. The site of stroke was classified using the Oxfordshire classification [20], which classifies stroke into total anterior circulation stroke (TACI), partial anterior circulation stroke (PACI), lacunar stroke (LACI), and posterior circulation stroke (POCI).

Cognitive status was measured with the Mini-Mental State Examination (MMSE) [16]. It tests orientation, memory, attention, calculation, language, and construction functions. Scores vary from 0 (severe cognitive impairments) up to 30 (no cognitive impairments). A score of less than 24 is considered as cognitive impairment.

The Barthel Index [21] measures the extent of independence and mobility in activities of daily living (ADL; i.e. feeding, bathing, grooming, dressing, bowel and bladder control, toileting, chair transfer, ambulation, and stair climbing). Scores range from 0 (completely dependent) up to 20 (completely independent).

In the Letter Cancellation Test [22], patients were requested to cross all ‘O’s (20 left, 20 right, 425 distractor letters) on a sheet of A4 paper. Both target and distractor letters were arranged in random order throughout the page. The difference in number of crossed letters on the contralesional and ipsilesional side was used to indicate VSN (i.e., an asymmetry between contralesional and ipsilesional sides of at least 2 omissions and hence, indicate VSN. Severity of VSN was defined as the magnitude of asymmetry in omissions between contralesional and ipsilesional sides (i.e. the larger the asymmetry in contra versus ipsilesional omissions, the more severe neglect).

Perception of upper paretic limb was tested with the Thumb Finding Test (TFT; [19], [23]). In this test the patient is asked with the eye closed to find his/her thumb with his unaffected hand, while the affected arm is positioned by the examiner in the lateral field front and eyes are closed. Scores vary from 0 (no deficit) up to 3 (unable to find the thumb).

#### Primary outcome measures

1. Motor part of the Fugl Meyer Arm Test (FM-arm; [24]) measures dependency in synergistic motor control of the upper paretic limb. FM-arm is a stroke-specific, performance based impairment index, designed to assess motor functioning and balance control in patients with post-stroke hemiplegia. It contains 57 items scored on a 3-point scale (i.e. 0, 1, and 2 points), measuring arm function (33, items, 0–66 points), leg function (17 items, 0–34 points), and balance (7 items, 0–14 points), with a maximum score of 114. Here, only the arm function was evaluated.
2. Motricity Index of the Arm (MI-arm; [25]) assesses strength of the upper paretic limb in stroke patients. There are three items for the arms (i.e. pinch grip, elbow flexion, shoulder abduction) as well as three items for the legs (i.e. ankle dorsiflexion, knee extension, hip flexion). Scores range from 0–100 (ordinal 6-point scale (i.e. 0, 11, 19, 22, 26, and 33 points) per item +1) for arms and legs separately. Here, only the three items for the arms were evaluated.

All clinical investigation has been conducted according to the principles expressed in the Declaration of Helsinki. All measurements were done by one investigator (GK) who was not involved in the patients' care and who was unaware of the assignments of the patients to the various rehabilitation groups. The study was approved by the institution's Ethics Review Board of VU University medical Centre, Amsterdam, the Netherlands. Participants gave written informed consent. When written consent could not be obtained from the patient directly, due to motor impairments, spoken informed consent of patients was required, after which the partner signed informed consent. The VU University medical Centre's Ethics Review Board approved of this procedure.

### Statistical analyses

The extent of functional neurological recovery explained by time was estimated for all primary outcome measures using random coefficient analysis. When the data structure in a population is hierarchical, sample data are viewed as a multistage sample from this hierarchical population [26]. Here, a hierarchical structure is clearly present as repeated observations (level 1) are nested within patients (level 2). The analysis of such data requires the implementation of multilevel statistical methods (e.g. random coefficient analysis) that account for the nesting of serial data within each subject. Nesting generates data that are correlated and statistically dependent. Multilevel models estimate regression coefficients and their related variance components while at the same time correct for the dependency of observations. Random coefficient analysis was performed with MLWin version 2.26 [26], [27], [28]. The iterative restricted generalized least-squares (IGLS) estimation procedure was used to estimate the regression coefficients of the derived model. The Wald-test was used to obtain p-values for a particular regression coefficient. We estimated the regression coefficients of three models. In the first model the regression coefficient was estimated for the association between the time-dependent predictor VSN severity (i.e. magnitude of asymmetry in omissions between contralesional and ipsilesional sides; an asymmetry ≤2 omissions is regarded as non-VSN, an asymmetry of >2 omissions is regarded as VSN [7]); and outcome (i.e. FM-arm or MI-arm). In the second model we investigated whether this relationship was time-dependent. Interaction terms (severity of VSN*time) were fitted to determine whether the post-stroke relationship between severity of VSN and outcome was dependent upon the time of measurement. If significant, the relationship between severity of VSN and outcome is not constant but becomes progressively stronger or weaker with each subsequent measurement in time. In the final model the latter time-dependent model is corrected for outcome baseline scores (i.e. FM-arm or MI arm baseline scores) to negate outcome differences at baseline. Consequently, we corrected the time-dependent relationship between FM-arm or MI-arm and VSN severity for FM-arm or MI-arm baseline scores, Oxfordshire classification score (reflecting severity of stroke), thumb finding test scores (reflecting sensory deficit) and administered rehabilitation program (to control for possible treatment effects). In the original study, patients were randomly assigned to a rehabilitation program with emphasis on either arm (30 min) or leg training (30 min), or 30-minute immobilization of the paretic arm and leg by an inflatable pressure splint (Svend Andersen, Haarlev, Denmark) within 7 days post-stroke. Each working day which served as the control group in the trial. All groups received 15 minutes per day leg training, 15 minutes per day arm rehabilitation, and 1.5 hours per week ADL training by an occupational therapist. For rehabilitation program, dummy variables were created with the control group as reference. For all tests, a two-tailed significance level of .05 was used.

Additionally, the exact timing of change in linearity of neurological recovery was measured using trend analyses, performed with JoinPoint Regression Program (Version 4.0.4, May 2013, Statistical Methodology and Applications Branch, Surveillance Research Program, National Cancer Institute; [29]). This software enables to test whether or not an apparent change in trend is statistically significant. Based on patterns of individual time series, JoinPoint fits the mean of trend data into the simplest log-linear function applicable. We investigated trends in FM-arm and MI arm recovery patterns over time. We tested whether these trends changed at some point in time for both groups separately and whether trends were similar for patients with and without VSN. Therefore, slope differences in trends as well the average weekly percent change (APC) were estimated and tested for difference from zero at alpha 0.05. Subsequently, a test of parallelism (mathematical similarity) was conducted to determine whether the two mean functions (longitudinal FM-arm and MI-arm mean scores) were parallel allowing different intercepts. Two mathematical functions are parallel if one function can be obtained from the other by a scaling of the dose axis. With a non-significant test result, the course of recovery of both groups is similar. Finally, a test of coincidence was conducted to determine whether the two mean functions (longitudinal FM-arm and MI-arm mean scores) were identical allowing different intercepts. With a non-significant result, the course of recovery of both groups coincide.

As multiple tests were performed, Bonferroni adjustment was used to ensure that the approximate overall type I error is less than the specified significance level.

## Results

During repeated assessments, 12 out of 101 stroke patients withdrew (six had recurrent stroke, two cancer, one carotid endarterectomy, two refused control treatment, and one died from a heart attack). Therefore, 1670 (92.3%) of the planned 1818 measurements were made in the present cohort. Mean time interval between stroke assessments was approximately 8 days.

### Demographics and stroke characteristics

An overview of demographics and stroke characteristics at baseline, split on VSN, is given in Table 1. There were no differences between groups at baseline with respect to age (U = 1163, p = .446), sex (χ^2^ (1)  = .099, p = .754), time post-stroke (U = 1142, p = .364), treatment condition (χ^2^ (2)  = .580, p = .748), and MMSE (U = 1023.5, p = .085). Groups differed with respect to the Barthel Index (U = 261.5, p = .008); Hemisphere (χ^2^ (1) = 16.990, p<.001); Site (χ^2^ (2)  = 29.883, p<.001); somatosensory deficit (χ^2^ (2)  = 8.335, p = .040); MI-Arm (U = 511, p = .005); MI-Leg (U = 537, p = .005); FM-arm (U = 292.5, p = .004); FM-leg (U = 352, p = .039). Of the patients with VSN, 9 patients showed right-sided VSN and 42 patients showed left-sided VSN.

### Random coefficient analysis

In Table 2, the means, variability and range of VSN severity (predictor) over time is presented.

**Table 2:** Means, variance, and range of VSN severity over time.

Time	Mean	Variance	Range
Baseline	4.18	25.31	19
Week 3	4.07	25.39	17
Week 4	3.40	21.74	17
Week 5	2.71	14.63	16
Week 6	3.14	19.81	18
Week 7	3.16	20.73	19
Week 8	2.73	13.10	16
Week 9	2.58	13.15	13
Week 10	2.76	17.80	19
Week 12	2.33	12.60	17
Week 14	2.54	16.59	18
Week 16	2.36	18.05	18
Week 18	2.24	13.77	18
Week 20	2.39	19.88	20
Week 26	2.49	18.23	19
Week 38	2.16	11.67	16
Week 52	2.38	14.78	18

#### FM-arm outcome

On average, lower FM-arm scores were associated with more severe VSN: an increase in VSN severity scores of 1 unit corresponded to a decrease of 0.34 in FM-arm scores (Table 3). However, this relationship was time-dependent (Table 4). During each additional measurement FM-arm scores increased with 0.69, while its relationship with VSN severity decreased with 0.03. Corrected for baseline and possible confounding variables, the FM-arm scores increased with 0.70 during each additional measurement, but its relation with VSN severity decreased with 0.03. In other words, an increase of VSN severity with *n* units (scale 0–20) coincides with FM-arm scores increase of (0.70–0.03*n), per measurement in time.

**Table 3:** Multilevel unstandardized regression coefficients, confidence intervals (CI) and level of significance for the association between VSN severity and time-dependent recovery of task (FM-arm and MI-arm) during the first year post-stroke.

Task	β value	CI	P-value
FM-arm			
* *Severity of VSN	−0.34	−0.44–0.24	<.001
MI-arm			
* *Severity of VSN	−0.79	−1.01–0.57	<.001

**Table 4:** Multivariate regression model: unstandardized regression coefficients, confidence intervals (CI) and level of significance for the analysis of the time-dependent association between VSN severity and task (FM-arm and MI-arm), corrected for type of treatment, outcome scores at baseline, severity of stroke, and sensory deficits, during the first year post-stroke.

Task	β value	CI	P-value
**FM-arm**			
Severity of VSN	0.15	0.01–0.29	.043
Time	0.70	0.63–0.76	<.001
Severity of VSN*time	−0.03	−0.04–0.02	<.001
FM-arm baseline	0.97	0.84–1.11	<.001
Type of treatment:			
Arm versus splint	4.95	1.79–8.12	0.002
Leg versus splint	2.08	−1.21–5.36	0.216
Severity of stroke	−6.22	−9.33–3.12	<.001
Sensory deficit	−0.90	−1.26–0.55	<.001
**MI-arm**			
Severity of VSN	0.24	−0.04–0.52	.093
Time	1.64	1.52–1.76	<.001
Severity of VSN*time	−0.06	−0.08–0.03	<.001
MI-arm baseline	0.89	0.78–1.00	<.001
Type of treatment:			
* *Arm versus splint	10.25	4.20–16.30	.001
* *Leg versus splint	5.62	−0.68–11.93	.081
Severity of stroke	−11.90	−17.83–5.98	<.001
Sensory deficit	−3.02	−6.06–0.02	.052

#### MI-arm outcome

Lower MI-arm scores were associated with more severe VSN: an increase in VSN severity scores of 1 unit corresponded to a mean decrease of 0.79 in MI-arm scores (Table 3). This relationship is also time-dependent (Table 4). During each additional measurement MI-arm scores increased with 1.62, while its relationship with VSN severity decreased with 0.05. Corrected for baseline and possible confounding variables, the MI-arm scores increased with 1.64 during each additional measurement, but its relationship with VSN severity decreased with 0.06. In other words, an increase of VSN severity with *n* units (scale 0–20) leads to MI-arm scores to increase with (1.64–0.06*n), per measurement in time.

### Changes in trends

#### FM-arm outcome

**Figure 1:**
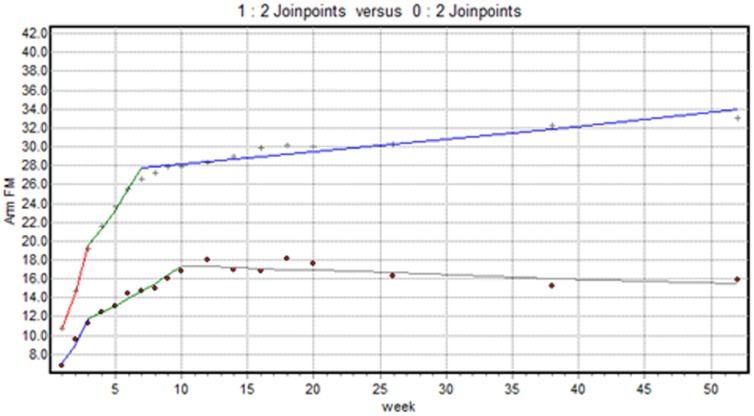
Observed changes in trends for the FM-arm scores of upper extremities between week 1 and week 52, split for VSN patients (dot) and non-VSN patients (+).

Figure 1 shows two significant trend changes in the pattern of recovery of motor synergism at 3 (p = 0.002) and 10 (p<0.001) weeks post-stroke in VSN patients. The largest recovery was observed within the first 3 weeks (APC1 = 29.64, CI = 15.9–44.9), followed by moderate recovery up to week 10 (APC2 = 5.76, CI = 3.8–7.8) and no recovery after 10 weeks (APC3 = −0.28, CI = −0.5– −0.1). For non-VSN patients, two significant trend changes in the pattern of recovery of synergism were found (both p<0.001). Largest recovery in the first 3 weeks (APC1 = 34.85, CI = 26.6–43.7), followed by moderate recovery up to 7 weeks post-stroke (APC2 = 9.20, CI = 5.8–12.7). Least significant recovery was found after 7 weeks (APC3 = 0.44, CI = 0.3–0.5). The test of coincidence was significant (p<.001), indicating that the regressions mean function was not identical. The test of parallelism was significant (p<.001), indicating that the regressions mean function was not parallel.

#### MI-arm outcome

**Figure 2:**
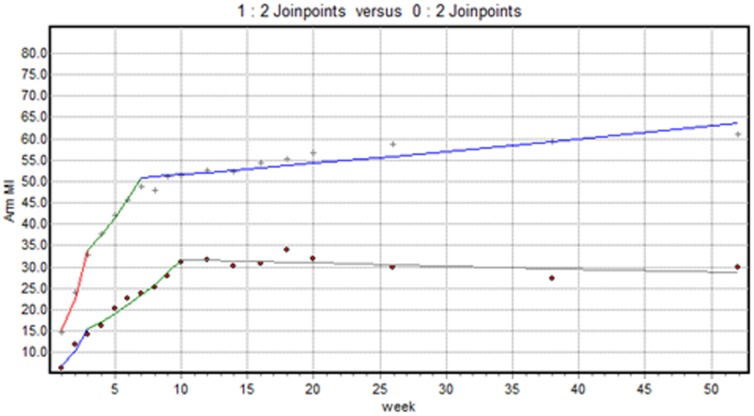
Observed changes in trends for the MI-arm scores of upper extremities between week 1 and week 52, split for VSN patients (dot) and non-VSN patients (+).

Figure 2 shows two significant trend changes in the pattern of motor strength were found for VSN at 3 (p = 0.003) and 10 weeks (p<0.001) post-stroke. The largest recovery was found in the first 3 weeks (APC1 = 51.99, CI = 27.2–81.5), moderate recovery up to week 10 (APC2 = 10.72, CI = 7.4–14.1) and no recovery after 10 weeks (APC3 = −0.24, CI = −0.6–0.1) post-stroke. For non-VSN patients, two significant changes in recovery were obtained, yet at different times post-stroke (p<0.001 and p = 0.002 respectively): the largest recovery was also obtained in the first 3 weeks (APC1 = 49.50, CI = 34.5–66.2), yet moderate recovery was found up to 7 weeks post-stroke (APC2 = 10.59, CI = 4.9–16.6), and least yet significant recovery after 7 weeks (APC3 = 0.50, CI = 0.3–0.7). The test of coincidence was significant (p<.001), indicating that the regressions mean function was not identical. The test of parallelism was significant (p<.001), indicating that the regressions mean function was not parallel.

## General Discussion

Currently, the critical period of spontaneous neurological change that contributes to observed cognitive, motor and activity recovery in the first months post-stroke is largely ignored in rehabilitation medicine [6], [7], [15], [30], [31], [32], [33]. The aim of the current study was to investigate the assumed remote, suppressive effects of VSN on the pattern of motor recovery of the upper paretic limb, as reflected by the Fugl-Meyer-arm score, during the first 52 weeks post-stroke. The focus was on FM-arm scores as they are often interpreted as a reflection of ‘true neurological repair’ by which patients regain their ability to control the different degrees of freedom in the paretic upper limb [11], [12]. Additionally, the impact of VSN on severity at stroke onset and recovery profiles was validated with Motricity Index of the arm (strength). Overall, the results suggested that more severe VSN is associated with more suppression on the pattern of recovery in synergism and strength from stroke-onset onwards resulting in slower improvements in time. However this association tapered off with each subsequent measurement in time. Trend analyses indicated that VSN patients not only have a significantly more severe impairment in strength at stroke onset, but also show less improvement in the first 10 weeks post-stroke when compared to non-VSN patients. Beyond this time window, further motor recovery was hampered in those with VSN when compared to non-VSN patients. With that, the suppressive effect of VSN on the pattern of motor recovery was mainly restricted to the same time-window in which spontaneous neurological recovery occurs [6]. Interestingly, not only the magnitude of motor recovery is much lower, the time course of recovery is also delayed and even becomes almost invariant after 10 weeks post-stroke onset.

Zarahn and colleagues [34] have indicated that there is substantial between-patient variation in recovery from upper limb impairment after stroke in patients with severe initial impairment. We included patients with severe motor impairment at start of the study; it is likely that variation in recovery occurs. As we analyzed both within and between subject variance, the current results are not a consequence of between-patient variation at onset, but also observed true differences in improvement within subjects that are associated with recovery of VSN.

Current findings suggest a common underlying mechanism of intrinsic neurological recovery, such as alleviation of diaschisis [13] in the first weeks, defining the time window within which certain magnitudes of improvement are expected to arise [6], [35]. There is also agreement with the hypothesis raised in the work of Feeny and Baron [13] suggesting that “diaschisis undergoes gradual regression in well-defined phases such that resolution will parallel resumption of function in areas of diaschisis.” One may assume that this alleviation of diaschisis reflects recovery of reduced metabolism of remote areas that are anatomically connected to the infarcted area. Probably, alleviation of diaschisis of remote areas may explain the observed spontaneous neurological changes early post-stroke [35]. The concept of functional cerebral distance of Kinsbourne and Hicks [36] might explain the hindering effect of VSN on magnitude of improvement in motor functions; when two tasks share processing resources, they are in close functional cerebral distance, which arises from significant anatomical interconnection between regions. In the case of (partial) damage to the motor cortex, residual motor cortex or more distant regions participate in the recovery process; the larger the damage, the more remote regions recruited to support recovery processes are. So far, functional distance or even the order in which regions will be recruited is unclear. One may hypothesize, however, that the suppressive effects of attentional networks [4], [37], [38] hamper spontaneous modulation of interhemispheric competition occurring in the first 10 weeks post-stroke onset, which will normally assist recovery of motor impairments as well as VSN in isolation [4], [39]. Future studies combining structural imaging (e.g. diffusion tensor imaging (DTI)) and behavioral techniques in an intensive repeated measurement design could disentangle the longitudinal association between neurological recovery and behavioral recovery and gain insight in the underlying mechanism as well.

Obviously, there are other factors that might influence recovery of motor functions as well. For example, it is known that VSN might foster non-use of the affected limbs in everyday life, which is likely to suppress mechanisms of spontaneous neurological recovery in the first three months post stroke [7] and probably negatively influence learning-dependent mechanisms of motor recovery on the affected hemiplegic side [4]. The decline of VSN allows patients to use the affected limbs more, which might benefit recovery as well. These complex interactions were not investigated in the current study. Additionally, the influence of mood changes, such as post-stroke depression might negatively affect recovery processes in general. This interaction was also not investigated in the current study.

As differences in both severity and functional outcome between left and right sided neglect patients have been suggested, it would have interesting to investigate differences in effects on motor recovery. The sample size of especially the group of right-sided neglect patients (n = 9) was too small, to statistically compare the time-dependent recovery patterns. Another limitation of the current study might be the use of the Oxfordshire classification as an approximation of severity of stroke. As no neuroimaging data is available, we cannot associate infarct size or volume, or even better integrity of white matter pathways to interactions between severity of VSN and recovery of upper limb impairment [40].

Future studies are needed to investigate whether repair or restitution of neurological deficits such as VSN is mainly restricted to the same time window in which spontaneous neurological recovery takes place. Recent kinematic studies, in which patients are measured from stroke onset onwards, show that the restitution of motor control by reducing jerk and controlling the degrees of freedom in reaching tasks are mainly defined within the first 5 to 8 weeks post stroke. This finding suggests that improvements in activities beyond this time window are mainly driven by adaptive motor strategies [30], [31], [33]. For example, recovery in performing activities such as wheel-chair navigation in patients suffering from VSN may be driven by spontaneous neurological recovery resulting in restitution of VSN function in the first weeks post-stroke, whereas after this time-frame patients gradually learn to deal with this neurological perceptual-attentional deficit. Obviously, both mechanisms contribute to observed improvement in requested functional tasks during recovery, however are based on different underlying mechanisms operating at different, sometimes overlapping time-frames post stroke [33]. As a consequence, the different training strategies aimed at restitution of substitution should be dependent on the moment post-stroke [33].

## Summary and Conclusions

This study is the first to disentangle the unique longitudinal courses of impaired motor functions patients with and without VSN as a function of progress of time. In the acute phase, VSN patients show more severe impairment of motor function and time-dependent recovery follows a different pattern with less improvement. Importantly, a suppressive, probably inhibitory effect of VSN on the pattern of improvement of motor impairment appears take place mainly within the first 10 weeks post-stroke, which is the exact same time-window in which spontaneous neurological recovery emerges.
